# +Psychometric evaluation of the MacDQoL individualised measure of the impact of macular degeneration on quality of life

**DOI:** 10.1186/1477-7525-3-25

**Published:** 2005-04-14

**Authors:** Jan Mitchell, James S Wolffsohn, Alison Woodcock, Stephen J Anderson, Carolyn V McMillan, Timothy ffytche, Martin Rubinstein, Winfried Amoaku, Clare Bradley

**Affiliations:** 1 Department of Psychology, Royal Holloway, University of London, Egham, Surrey, TW20 0EX, UK; 2 Neurosciences Research Institute, Aston University, Birmingham, B4 7ET, UK; 3 Hospital for Tropical Diseases, Capper Street, London WC1E 6AU, UK; 4 Eye Department, Queen's Medical Centre, Derby Road, Nottingham, NG7 2UH, UK

## Abstract

**Background:**

The MacDQoL is an individualised measure of the impact of macular degeneration (MD) on quality of life (QoL). There is preliminary evidence of its psychometric properties and sensitivity to severity of MD. The aim of this study was to carry out further psychometric evaluation with a larger sample and investigate the measure's sensitivity to MD severity.

**Methods:**

Patients with MD (n = 156: 99 women, 57 men, mean age 79 ± 13 years), recruited from eye clinics (one NHS, one private) completed the MacDQoL by telephone interview and later underwent a clinic vision assessment including near and distance visual acuity (VA), comfortable near VA, contrast sensitivity, colour recognition, recovery from glare and presence or absence of distortion or scotoma in the central 10° of the visual field.

**Results:**

The completion rate for the MacDQoL items was 99.8%. Of the 26 items, three were dropped from the measure due to redundancy. A fourth was retained in the questionnaire but excluded when computing the scale score. Principal components analysis and Cronbach's alpha (0.944) supported combining the remaining 22 items in a single scale. Lower MacDQoL scores, indicating more negative impact of MD on QoL, were associated with poorer distance VA (better eye r = -0.431 p < 0.001; worse eye r = -0.350 p < 0.001; binocular vision r = -0.419 p < 0.001) and near VA (better eye r = -0.326 p < 0.001; worse eye r = -0.226 p < 0.001; binocular vision r = -0.326 p < 0.001). Poorer MacDQoL scores were associated with poorer contrast sensitivity (better eye r = 0.392 p < 0.001; binocular vision r = 0.423 p < 0.001), poorer colour recognition (r = 0.417 p < 0.001) and poorer comfortable near VA (r = -0.283, p < 0.001). The MacDQoL differentiated between those with and without binocular scotoma (U = 1244 p < 0.001).

**Conclusion:**

The MacDQoL 22-item scale has excellent internal consistency reliability and a single-factor structure. The measure is acceptable to respondents and the generic QoL item, MD-specific QoL item and average weighted impact score are related to several measures of vision. The MacDQoL demonstrates that MD has considerable negative impact on many aspects of QoL, particularly independence, leisure activities, dealing with personal affairs and mobility. The measure may be valuable for use in clinical trials and routine clinical care.

## Background

Macular degeneration (MD) is a chronic, progressive eye condition that mainly affects people over the age of 50 years. It is the leading cause of blindness among those of European descent over the age of 60 years [1]. Recently it was estimated that, in the UK, between 182,000 and 300,000 people are blind or partially sighted because of MD [2]. For the majority there is no treatment and, where treatment is available, it does not cure the condition but instead slows or halts its progress for an indeterminate period [3]. People with MD lose their central vision and this precludes daily activities requiring fine vision such as reading, driving, watching TV and recognising faces. Peripheral vision is usually retained. MD can impair efficiency in performing most daily activities and may compromise the ability to live an independent life. The psychological impact of the condition can be devastating [4,5]. An ageing population means that the prevalence of MD is likely to increase [3].

New treatments for MD are being developed, as are rehabilitation programmes. Quality of life (QoL) is increasingly required as an outcome measure in clinical trials and an appropriate instrument is necessary. There has been little consensus about the definition and measurement of QoL in ophthalmology, just as in other areas of medicine [6]. Measures of health status, functional status and psychological well-being have all been used and described as QoL measures, but the interpretation of data used in this way can be misleading [7]. Some researchers into the impact of vision impairment on QoL have used health status measures such as the SF-36 [8] or the Sickness Impact Profile [9], but these have not proved informative [10,11], as many of the aspects of 'health' investigated in generic measures are unlikely to be affected by MD. Others have measured functional status (e.g. activities of daily living) [12], referring to it as QoL. Measures of health status and functional status do not correlate well with visual acuity (VA). Self-reported visual function, investigated using measures such as the NEI-VFQ [13] or the Activities of Daily Vision Scale [14] is moderately associated with VA. While such instruments can provide valuable information about functional impairment caused by vision loss, they do not measure the impact on QoL. One useful way of measuring the impact of an eye condition on QoL is to consider the importance to individuals of the aspects of life investigated in the questionnaire as well perceptions of the impact of their eye condition on each aspect. The principle of including participants' ratings of the importance of domains to their QoL (by ranking the domains) has been adopted in some generic QoL measures including the SEIQoL [15] and the Patient Generated Index [16].

**Figure 1:**
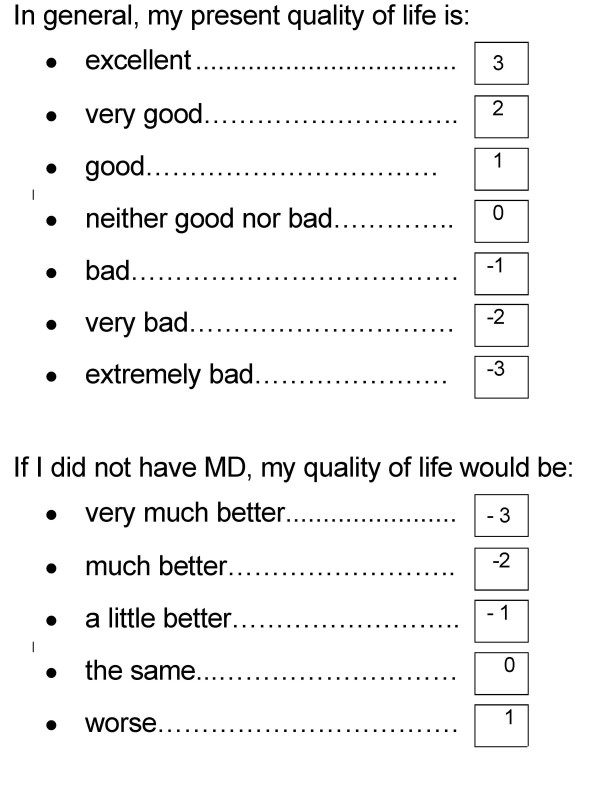
MacDQoL present QoL and MD-specific overview items with scores shown.

**Figure 2:**
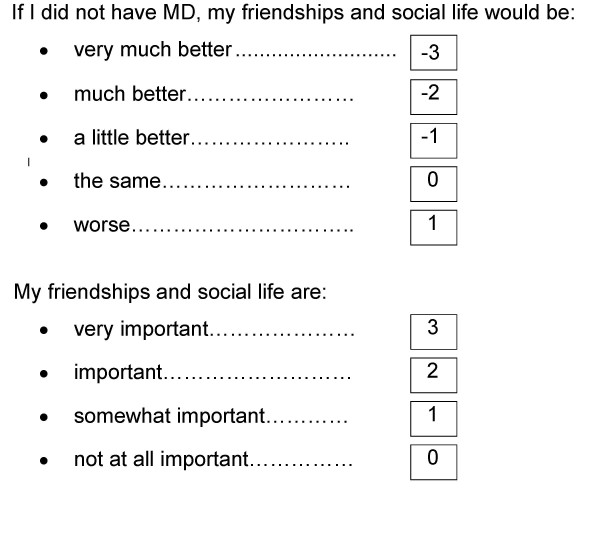
MacDQoL domain-specific item with scores shown.

The MacDQoL is an individualised measure of the impact of MD on QoL, based on the design of the Audit of Diabetes Dependent Quality of Life (ADDQoL) [17], which is increasingly used [18-20]. The questionnaire begins with two overview items, measuring: a) present QoL. (*In general, my present quality of life is:*), scored from +3 (*excellent*), through 0 (*neither good nor bad*) to -3 (*extremely bad*), b) MD-specific QoL (*If I did not have MD, my quality of life would be:*), scored from -3 (*very much better*) through 0 (*the same*) to +1 (*worse*). The 26 domain-specific items in the MacDQoL were developed from focus group meetings with people who have MD and with reference to the literature and to psychologists experienced in this field (Table 2) [21]. Each has questions asking about both the impact of MD on that aspect of life and the importance of the aspect of life to QoL. The paper version is designed for completion by visually impaired people. Figures 1 and 2 show the presentation in the questionnaire of the two overview items and one domain-specific item, with the scores for each response option shown. For the domain-specific items, impact scores (from -3 to +1) are multiplied by importance scores (from 0 to 3) to give a weighted impact score for each domain of between -9 and +3. The use of impact and importance scores enables an estimation of the impact of MD on an individual's QoL, not merely on function. For example, MD may adversely affect the time it takes an individual to do things, but if time taken is not important to his/her quality of life there will be no negative impact on QoL. Conversely, a small impact on a domain such as family life may lead to a considerable diminution of QoL if family life is very important to a person. Some domains have a 'not applicable' option (indicated by *, Table 2). A final item asks the respondent whether MD affects his/her life in any ways not already covered by the questionnaire, with a space to write a response for people who reply 'yes'. The measure has face and content validity and preliminary evidence of internal consistency reliability and sensitivity to differences in vision status (registered as blind, partially-sighted or not registered) has been reported previously [21]. Other work has shown preliminary evidence of reproducibility using self-completion in a sample of 61 people with MD [22]. The correlation between scores at time one and time 2 (mean interval 39 days) was 0.9 and there was no difference between AWI scores at times one and two (t = 1.2, p > 0.05).

The research reported here formed the first part of a longitudinal study to carry out further evaluation of the MacDQoL.

Previous research has indicated that completion of vision-related questionnaires by pen and paper (self-completion) and by interview may not yield equivalent results [23]. This is also the case for the MacDQoL [22]. We anticipated that a substantial proportion of participants in this study would be unable to self-complete the MacDQoL because of their visual impairment and it was decided to complete the measure by telephone interview for all participants.

## Methods

### Participants

Potential participants were identified from the clinic lists (NHS and private) of a consultant ophthalmologist (WA). Patients were considered suitable if they had age-related MD, treated or untreated, in one or both eyes. They were excluded for any of the following:

• cataracts that were considered sufficiently severe to impair vision

• glaucoma

• diabetic retinopathy sufficiently severe to impair vision

• degenerative myopia

• any macular condition other than age-related MD

• one non-functioning eye for reasons other than age-related MD

• unable to understand and speak English

### Procedure

Patients who met the inclusion criteria were contacted, initially by telephone, by an ophthalmic nurse known to all the patients. She told patients about the research, reading from a prepared script, and invited them to participate. Those willing to take part were given an appointment for a vision assessment at the hospital. Written information was despatched within three days of the telephone conversation. A member of the research team (JM) telephoned soon after and agreed the time of a telephone interview, which was carried out by a psychologist (CM or JM) not more than 14 days prior to the vision assessment appointment. During the interviews participants completed:

• MacDQoL

• demographic items

• other vision-related questions followed the MacDQoL and the demographic items. These will be reported fully elsewhere.

Interviewers were not informed of the clinical characteristics of the individual participants

Responses to questions were entered into a computerised on-screen questionnaire using SPSS Data Entry Builder [24]. The data were automatically stored as an SPSS data file.

Vision assessments, carried out by optometrists (SA, JW, MR) included:

• distance visual acuity, using Bailey-Lovie logMAR charts with Early Treatment for Diabetic Retinopathy Study (ETRDS) protocol [25] for monocular and binocular vision

• near visual acuity (MNREAD charts with ETDRS protocol) for monocular and binocular vision [26]

• comfortable visual acuity for monocular and binocular vision. This was computed from time taken to read script of different sizes of print (MNREAD charts with ETDRS protocol). The time taken to read each line was recorded. When the time to read a line increased substantially, this showed that it was no longer 'comfortable' to read that size print and smaller prints [26].

• contrast sensitivity, using Pelli-Robson charts [27] for monocular and binocular vision

• presence of distortion or a scotoma in central 10 degrees of vision (Amsler grid with concentric circles) for monocular and binocular vision. Patients fixated the central spot and identified the presence of distorted or missing grid lines in their peripheral field [28].

• colour vision (PV-16 colour vision test for visually impaired people) for binocular vision only. This consisted of a number of coloured blocks that the participant was asked to arrange in the order of the spectrum and is an enlarged version of the D-15 colour vision test [29].

• recovery from glare (Eger stressometer glare test) for binocular vision only. This test recorded the number of seconds taken to be able to read the smallest readable print again, after a brief flash of light [30].

The optometrists who carried out the vision assessments were not provided with participants' questionnaire responses.

These data were entered manually into Excel and transferred to SPSS.

Ethical approval was obtained from the Nottingham Research Ethics Committee.

### Statistical methods

SPSS 9.0 [31] was used. The range of responses was examined to ascertain the need for the full range of response options and the 'not applicable' options. The effect of incorporating impact and importance ratings on the rank order of domains was investigated.

Fourteen of the 26 MacDQoL domain-specific items had a non-normal distribution. Since reliability and factor analyses are parametric procedures, measures were taken to normalise the data using transformations. Principal components analyses were carried out on both raw and transformed data.

### Factor structure

Principal components analysis was carried out to identify possible subscales within the MacDQoL. To allow for data from the maximum number of participants to be used in the psychometric analyses, principal components analysis and internal consistency reliability analyses were conducted twice: first with missing data due to items being not applicable recoded as zero and participants with missing data being deleted listwise; secondly with 'not applicable' responses treated as missing data and pairwise deletion being used to minimise loss of data.

### Internal consistency reliability

Cronbach's alpha coefficient of internal consistency reliability of was calculated. The higher the alpha, the stronger the internal consistency reliability, indicating that all items are measuring aspects of the same underlying construct. Corrected item-total correlations were carried out to investigate the strength of individual items' associations with the construct.

### Redundancy

Redundancy of items was investigated by examining correlations between items. The distributions of the scores of the items were examined and Wilcoxon signed rank tests were carried out to compare scores of items of similar content. Principal components analysis and Cronbach's alpha were repeated after removal of redundant items.

### Construct validity

Construct validity is established by examining predicted relationships between the questionnaire scores and other clinical or psychological variables. Spearman's correlations and Mann Whitney tests were used to investigate the relationship between the MacDQoL overview items and the average weighted impact score (AWI) with twelve measures of vision (see Table 6).

It was hypothesised that the MD-Specific QoL overview item and the AWI would be correlated with better eye and binocular distance visual acuity (VA), better eye and binocular near VA, better eye and binocular contrast sensitivity, binocular colour recognition, binocular comfortable reading speed and binocular presence or absence of scotoma and distortion, with greater visual impairment being associated with greater impact of MD on QoL. Since it does not focus specifically on the impact of MD on QoL, it was also hypothesised that the present QoL overview item would be correlated with these variables, but less strongly than the MD-Specific QoL overview item and the AWI.

## Results

### Participants

Of the 223 people telephoned by the research nurse, 38 people (17%) declined to take part (mean age of those who declined = 79.8 ± 13 years, 47% women, 53% men). Reasons for non-participation included being too ill, having too far to travel to the hospital or being unable to make suitable travel arrangements, having no one to accompany them to the vision assessment, being unavailable on the vision assessment dates and not being interested in taking part in the research. Twenty-nine people (69% women, 31% men, mean age 82.6 years) who agreed initially to take part subsequently changed their minds, or did not attend the vision assessments for other reasons. Of these, five completed the telephone interview before deciding not to participate further.

The mean age of the 156 participants was 78.96 years (s.d. 6.64, median 79.76, range 52.47 to 91.61). The mean age at leaving full time education was 15.28 years (s.d. 2.21, median 14 years, minimum 12 years, maximum 27 years). Other demographic data are reported in Table 1.

**Table 1:** Patient characteristics: Sex, marital status, number of eyes affected by MD, type of MD, whether both eyes diagnosed at same time, registration status.

Demographic and clinical data		N (valid %)
Sex	women	99 (63.5)
	men	57 (36.5)
Marital status	married or living with partner	74 (47.4)
	widowed	68 (43.6)
	divorced or separated	8 (5.1)
	single	6 (3.6)
Number of eyes affected by MD	one	6 (3.8)
	two	150 (96.2)
Type of MD	wet only	90 (57.7)
	dry only	19 (12.2)
	wet and dry	42 (26.9)
	wet and type MD in 2^nd ^eye not specified	4 (2.6)
	type of MD not specified	1 (0.6)
Both eyes diagnosed at same time	yes	46 (32.6)
	no	95 (67.4)
	missing	15
Registration status	blind	8 (5.4)
	partially sighted	67 (45.6)
	not registered	72 (46.2)
	missing	9

**Table 2:** Frequencies of impact and importance scores for domains of the MacDQoL

**Item**	**Impact score frequencies**					**Importance score frequencies**			
	** -3**	** -2**	** -1**	** 0**	** 1**	** 3**	** 2**	** 1**	** 0**

household tasks	46	56	26	28	0	55	76	20	5
personal affairs	65	41	22	28	0	75	57	17	6
shopping	67	43	24	22	0	47	76	23	10
*work	1	2	0	0	0	1	1	1	0
*personal relationship	10	12	12	47	0	58	19	3	0
*family life	27	35	24	60	3	108	36	4	1
friends and social	33	43	26	54	0	63	71	14	8
physical appearance	22	22	41	71	0	67	57	26	6
do physically	44	50	37	25	0	80	62	12	2
get out and about	62	34	30	30	0	92	50	12	2
*long journeys	28	37	13	24	1	27	41	26	10
*holidays	38	33	23	27	0	38	48	27	8
leisure activities	97	37	16	13	0	64	65	21	6
hobbies	68	46	19	22	0	63	64	23	6
self-confidence	41	55	29	31	0	80	58	12	6
motivation	31	48	31	45	1	51	64	33	8
people's reaction	12	25	27	91	0	48	66	27	14
society's reaction	14	25	26	88	0	28	61	43	22
future	43	56	27	30	0	52	60	33	11
financial situation	11	13	12	119	1	38	79	30	9
independence	71	37	27	21	0	97	45	10	4
do for others	56	46	23	31	0	66	65	21	4
mishaps	40	34	43	39	0	69	60	21	6
enjoy meals	31	33	26	66	0	49	72	26	9
time taken	41	49	32	33	0	30	56	45	24
enjoy nature	48	47	23	38	0	56	62	26	12

* indicates a 'not applicable option

Clinical data are reported in Table 1. Only six (3.8%) people had just one eye affected by MD. Ninety people (57.7%) had wet MD in both eyes.

### The MacDQoL: range of responses

The completion rate for the MacDQoL items was 99.8%

The full range of scoring options for impact of MD (-3 to +1) was used in four domains (Table 2). All scoring options except +1 (indicating a positive impact of MD on QoL) were used in all other domains except *work*, where only -2 and -3 were used. The most negatively impacted domain in the MacDQoL was *work *(-2.33), although this domain was applicable to only three respondents (Table 3). Among the least impacted domains were *finances *(-0.45) and *people's reaction *(-0.73) (Table 3).

**Table 3:** MacDQoL domain-specific items in descending order of impact; mean impact scores, mean importance scores and positions of domains in rank order of weighted impact

**Domains in descending order of impact score (n)**	**Mean impact score (s.d.)**	**Mean importance rating (s.d.)**	**Rank order of weighted impact**
1 work (3)	-2.33 (1.08)	2 (1)	3
2 leisure activities (155)	-2.31 (0.96)	2.2 (0.81)	2
3 hobbies (156)	-2.03 (1.07)	2.18 (0.82)	4
4 independence (156)	-2.01 (1.08)	2.51 (0.73)	1
5 shopping (156)	-1.99 (1.07)	2.03 (0.84)	9
6 personal affairs (156)	-1.92 (1.13)	2.3 (0.82)	5
7 get out and about (156)	-1.82 (1.16)	2.49 (0.7)	6
8 do for others (156)	-1.81 (1.13)	2.24 (0.78)	7
9 household tasks (156)	-1.77 (1.06)	2.16 (0.77)	12
10 do physically (156)	-1.72 (1.04)	2.41 (0.69)	8
11 future (156)	-1.72 (1.07)	1.98 (0.91)	13
12 self-confidence (156)	-1.68 (1.07)	2.36 (0.79)	11
13 holidays (121)	-1.68 (1.14)	1.96 (0.9)	14
14 nature (156)	-1.67 (1.15)	2.04 (0.91)	10
15 long journeys (103)	-1.65 (1.14)	1.82 (0.93)	17
16 time taken (156)	-1.63 (1.09)	1.59 (0.97)	19
17 mishaps (156)	-1.48 (1.13)	2.23 (0.83)	15
18 motivation (156)	-1.40 (1.12)	2.01 (0.86)	20
19 friends and social (156)	-1.35 (1.16)	2.21 (0.81)	18
20 enjoy meals (156)	-1.19 (1.18)	2.03 (0.85)	21
21 family life (149)	-1.15 (1.2)	2.68 (0.56)	16
22 physical appearance (156)	-1.14 (1.08)	2.19 (0.85)	22
23 personal relationship (81)	-0.81 (1.10)	2.69 (0.54)	23
24 society's reaction (153)	-0.77 (1.03)	1.62 (0.94)	25
25 people's reaction (155)	-0.73 (1.00)	1.95 (0.92)	24
26 financial situation (156)	-0.45 (0.92)	1.94 (0.82)	26

**Figure 3:**
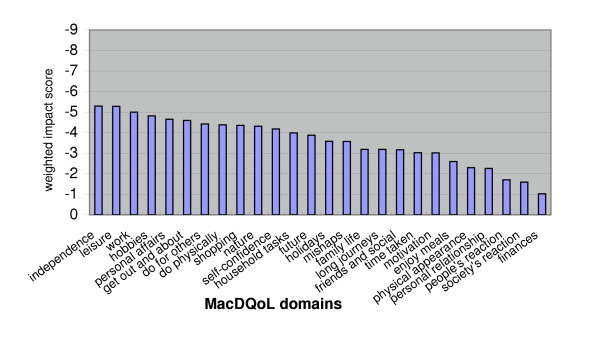
Mean weighted impact scores of MacDQoL domains.

The full range of importance ratings (0 – 3) was used in 24 of the 26 domains (Table 2). Mean importance ratings ranged from 2.69 (*personal relationship*) to 1.59 (*time taken*) (Table 3). The rank order of domains in order of impact score changed when impact scores were multiplied by importance to give weighted impact scores, with only three domains remaining in the same position after weighting by importance (Table 3). Positions in the rank order of mean values changed by between zero and five places. Changes for individual respondents were even more substantial. Figure 3 shows the weighted impact scores of each domain. The greatest negative impact was reported for *independence *(-5.29) followed by *leisure *and *work*. The least impacted domain was *finances *(-1.02).

Five items had not applicable (N/A) options (Table 2). The greatest use of the N/A option was for *work *(n = 153, 98%), followed by *personal relationship *(n = 75, 48%). Only seven (4.5%) people reported that *family life *was N/A.

### Transforming the data

Data for some MacDQoL domains were not normally distributed. Average weighted impact scores were transformed using first log and then reflect and log transformations. It was not possible to achieve normality for all domains using either transformation, though the size of the sample will protect against the problems of non-normality. Principal components analyses using transformed data produced results that were very similar to those using untransformed data. The results reported here were obtained using untransformed data.

### Structure of 26-item MacDQoL

#### (a) Not applicable items scored as zero

Principal components analysis with varimax rotation produced five components with Eigenvalues greater than 1. Eleven items loaded >0.4 on the first factor, including items relating to activities, such as *household tasks*, *personal affairs*, *getting out and about*, *hobbies *and *do things for others*. Eight items loaded on the second factor, including several relating to self-consciousness, such as *appearance*, *people's reaction *and *mishaps*. *Finances *loaded on factors 2 and 5 and *leisure *and *hobbies *loaded on both factors 1 and 3. In a forced single-factor analysis, all items loaded > 0.4 except *work *and *finances*.

#### (b) Not applicable items scored as missing, using pairwise deletion

*Work *was removed from the analysis because it was applicable for only three people. Principal components analysis with varimax rotation seeking Eigenvalues >1 revealed a 4-factor structure. Seven items double-loaded and the factor structure and the factors were not conceptually distinct. A forced single-factor analysis showed loadings very similar to the one with N/A scored as 0 except that *personal relationship *loaded 0.662 with N/A scored missing compared with 0.419 with N/A scored as zero. *Finances *still loaded < 0.4.

#### (c) Removal of items

A priority was to shorten the questionnaire to reduce the demand on respondents. Three pairs of items were investigated to establish whether there was any redundancy: *People's reaction *and *society's reaction*; *leisure *and *hobbies*; *holidays *and *long journeys*.

The items *society's reaction *and *people's reaction *were originally both included to establish which one was easier to understand. The telephone interviewers found that participants hesitated less over *people's reaction *and sometimes had difficulty differentiating between the two items. The item scores were highly correlated with each other (r = 0.692, p <0.001), more so than with any other items. The distributions of impact and importance scores were similar for the two items. To control for familywise error, a Bonferroni correction was applied (p < 0.016 accepted). There was no difference in weighted impact scores between the two items (median *people's reaction = *0 [range 0 to -9]; median *society's reaction *= 0 [range 0 to -9], p > 0.05). *People's reaction *is easier to translate into other languages and this is an important consideration if the measure is to be used in international trials. Finally, evidence from semi-structured interviews in the UK and Germany during the development of a similar measure for use in diabetic retinopathy (RetDQoL) [32] supported the inclusion of *people's reaction *rather than *society's reaction *on grounds of ease of comprehension. So *ciety's reaction *was therefore dropped and *people's reaction *retained.

The items *leisure activities *and *hobbies and interests *were highly correlated with each other (r = 0.711, p < 0.001). Distribution of scores was similar for the two items. A Wilcoxon signed ranks test showed no significant difference after applying the Bonferroni correction (median *leisure activities *= -6 [range 0 to -9], median *hobbies and interests *= -6 [range 0 to -9]; Z = -2.33, p = 0.02; p < 0.016 accepted). The telephone interviewers noted that people often talked about hobbies and other interests such as embroidery and playing musical instruments when considering the *leisure *item. The understanding of these two items appeared to overlap and retaining both may lead to artificial inflation of the AWI. Therefore only one item was retained and reworded to specify leisure activities as well as hobbies. For the purposes of analysing the present data the mean of the two weighted impact scores was calculated for each participant (hobbies and leisure = [hobbies_wi _+ leisure_wi_]/2).

The items *long journeys *and *holidays *were highly correlated with each other (r = 0.692, p <0.001). The patterns of distribution of the scores were similar for both items. There was no difference in weighted impact scores for the two items (median *long journeys *= -3 [range 3 to- -9], median *holidays *= -3 [range 0 to -9]; Z = -1.82, p > 0.05). Fewer scores were lost to the N/A option with *holidays *than with long *journeys*. During telephone interviews, the earlier item, *long journeys*, elicited comments about *holidays*, and respondents often considered the two activities to be part of the same event, since most people were retired and so work-related travel was not a consideration. To keep both items may lead to artificial inflation of the AWI, so *holidays *was retained and *long journeys *removed.

#### (d) Structure of the 23-item MacDQoL

Further principal components analyses were carried out following the removal of the three items. An unforced analysis with varimax rotation yielded four factors. The first factor still contained predominantly activity items together with *confidence*. The remaining three factors could not be labelled coherently. *Appearance *did not load on to any factor >0.4. In a forced single-factor analysis, all items except *work *and *finances *loaded > 0.42.

*Work *was removed and the analyses re-run, with N/A items scored as zero. Again, principal components analysis yielded four factors (Table 4). Six items double-loaded and one of the factors was not conceptually distinct. In a forced one-factor analysis of the 22 items, all items loaded >0.42, except *finances*, although the loading of this now approached 0.4 (0.356)(Table 4). The item *work *was applicable to only three people, but those for whom it was applicable reported a high negative impact. It was decided that *work *should remain in the questionnaire, but be scored as a separate item. The weighted impact score of *finances *was the lowest of all remaining 22 items, at -1.02. However, some negative impact of MD on *finances *was reported by 35 (23%) of participants and only nine (5.8%) people thought it was not at all important. It was decided to retain *finances*, not only because of the relevance to a minority in the present UK sample but also because this aspect of life is likely to be more impacted in people from countries where there is greater financial hardship for people with vision loss due to MD.

**Table 4:** Unforced principal components analysis with varimax rotation after removal of items and forced one-factor analysis with N/A items scored as zero (items loading at > 0.4 in bold).

Item	Rotated Component Matrix Four factor solution (variance explained = 64.3%)				Single factor solution (variance explained = 49%)
	Factor 1	Factor 2	Factor 3	Factor 4	Factor 1
household tasks	**0.734**	0.098	0.136	0.312	**0.6869**
personal affairs	**0.754**	0.254	0.146	0.099	**0.7264**
shopping	**0.747**	0.042	0.317	0.219	**0.7145**
personal relationship	0.210	-0.062	0.132	**0.778**	**0.4219**
family	0.179	** 0.411**	0.199	**0.629**	**0.6127**
friends and social	0.252	** 0.604**	0.095	**0.541**	**0.6961**
appearance	0.342	0.210	**0.443**	0.181	**0.5821**
do physically	**0.587**	** 0.473**	0.269	0.023	**0.7659**
get out and about	**0.699**	0.324	0.252	0.125	**0.7809**
holidays	0.199	** 0.793**	0.188	0.050	**0.6582**
hobbies/leisure	**0.589**	** 0.549**	0.081	0.091	**0.7484**
self-confidence	**0.495**	0.282	0.259	0.266	**0.6691**
motivation	0.387	** 0.444**	0.310	0.291	**0.7150**
peoples reaction	0.265	0.259	**0.702**	-0.071	**0.5935**
future	0.189	0.284	**0.533**	0.162	**0.5541**
finances	0.048	-0.070	**0.747**	0.148	0.3558
independence	**0.716**	** 0.444**	0.173	0.158	**0.8345**
do for others	**0.679**	0.322	0.227	0.099	**0.7491**
mishaps	0.387	** 0.651**	**0.419**	0.176	**0.7906**
enjoy meals	0.375	0.349	**0.538**	0.160	**0.7141**
time taken	0.321	** 0.460**	**0.466**	0.204	**0.7208**
nature	0.305	** 0.752**	0.150	0.127	**0.7122**

### Reliability of the 22-item MacDQoL AWI scale score

Internal consistency reliability of the shortened, 22-item scale was investigated, first with N/A items scored as zero (N = 151). Cronbach's alpha coefficient of internal consistency reliability was 0.944. When the analysis was repeated with N/A items scored as missing (N = 62), alpha was 0.946. In both cases only *finances *detracted from the reliability, reducing it negligibly, by 0.012 in each case. The pattern of results was similar for both methods of dealing with N/A items. Table 5 shows the reliability analysis with N/A scored as zero.

**Table 5:** Reliability of the 22-item MacDQoL scale, with N/A items scored as zero (Cronbach's alpha = 0.9440)

MacDQoL item	Scale mean if item deleted	Scale variance if item deleted	Corrected item-total correlation	Alpha if item deleted
household tasks	-71.92	1890.0	0.65	0.9414
personal affairs	-71.19	1855.3	0.68	0.9408
shopping	-71.53	1876.8	0.68	0.9410
personal relationship	-74.74	1967.5	0.39	0.9446
family	-72.93	1879.3	0.58	0.9426
friends and social	-72.80	1875.0	0.66	0.9412
appearance	-73.65	1915.5	0.54	0.9428
do physically	-71.57	1858.6	0.72	0.9402
get out and about	-71.32	1838.4	0.74	0.9399
holidays	-73.16	1882.9	0.61	0.9419
hobbies/leisure	-70.87	1874.7	0.71	0.9405
confidence	-71.66	1879.3	0.63	0.9417
motivation	-72.83	1876.9	0.68	0.9409
people's reaction	-74.16	1918.8	0.55	0.9427
future	-72.03	1909.2	0.52	0.9433
financial situation	-74.89	1987.8	0.33	0.9452
independence	-70.57	1822.7	0.80	0.9390
do for others	-71.45	1847.3	0.71	0.9404
mishaps	-72.31	1839.5	0.76	0.9397
meals	-73.30	1882.1	0.68	0.9410
time	-72.88	1870.7	0.69	0.9408
nature	-72.08	1866.9	0.67	0.9410

**Table 6:** Mean scores for MacDQoL variables and vision measures. For distance VA and near VA, larger numbers indicate poorer vision. For contrast sensitivity, larger numbers indicate greater sensitivity. Larger numbers indicate poorer colour recognition and comfortable VA. For the glare test, larger numbers indicate a longer recovery time.

Variable		Mean	s.d.	Median
MacDQoL present QoL overview		0.90	1.13	1
MacDQoL MD-specific overview		-2.13	0.89	-2
MacDQoL AWI		-3.57	2.14	-3.7
				
Distance VA (logMAR)	better eye	0.42	0.46	0.39
	worse eye	1.23	0.95	0.95
	binocular	0.39	0.46	0.37
Near VA	better eye	0.45	0.43	0.3
	worse eye	1.09	0.58	1.1
	binocular	0.42	0.41	0.30
Contrast sensitivity	better eye	1.01	0.77	1.05
	worse eye	0.43	0.49	0.15
	binocular	1.01	0.44	1.05
Comfortable VA		0.50	0.35	0.4
Colour recognition (errors)		21.60	7.74	21.9
Glare test recovery (seconds)		11.02	8.94	8.5

### Missing data

The AWI score can be computed despite some missing data. Missing data for up to half the items can be tolerated without Cronbach's alpha falling below 0.8. The AWI score can be calculated from the items for which responses have been given providing at least 11 items have complete responses.

### Correlation between MacDQoL AWI and overview items

Mean scores of the MacDQoL overview items and AWI scores are shown in Table 6. Spearman's r correlations indicated that the AWI score was, as expected, more highly correlated with the MD-specific QoL overview item (r = 0.58, N = 156, p < 0.001) than with the present QoL item (r = 0.47, N = 156, p < 0.001).

### Construct validity

Construct validity of the MacDQoL was investigated by examining relationships between the two overview items and AWI scores and the twelve measures of vision taken at the visual assessments. Since the MD-specific overview item and several of the vision measures yielded non-normal data, non-parametric tests were used.

Mean scores of the vision measures for better and worse eye and binocular vision are shown in Table 6. Spearman's correlations were used to investigate relationships between these and the three MacDQoL variables (Table 7). To control for the possibility of familywise error with 36 correlations, a Bonferroni correction was applied (p < 0.00138 accepted). Twenty-nine of the 36 correlations indicated associations of poorer QoL with worse vision, with p-values of <0.05. Twenty of these associations were still significant after correcting for familywise error (p < 0.00138). As expected, in most cases, the AWI score correlated with vision measures more strongly than did the two overview items. For near VA, distance VA and contrast sensitivity, the strongest correlations were with better-eye scores as predicted. Binocular measures showed similar relationships and worse eye measures showed poorer and less consistent associations with the MacDQoL variables Table (7). Comfortable VA and colour recognition were not associated with present QoL and comfortable VA was not associated with the MD-specific QoL overview item. None of the three MacDQoL variables was associated with recovery from glare Table (7), neither were relevant individual items, such as *holidays *or *get out and about*.

**Table 7:** Correlations (Spearman's r) between MacDQoL outcome variables and vision measures (*remains significant after Bonferroni correction).

		Present QoL	p-value	MD-specific QoL	p-value	AWI	p-value
Distance VA	better eye	-0.301	<0.001*	-0.310	<0.001*	-0.431	<0.001*
Near VA	better eye	-0.327	<0.001*	-0.192	0.017	-0.326	<0.001*
Contrast sensitivity	better eye	0.200	0.012	0.300	0.001*	0.392	<0.001*
Colour vision	binocular	-0.204	0.011	-0.291	<0.001*	-0.417	<0.001*
Comfortable VA	binocular	-0.207	0.012	-0.121	>0.05	-0.283	<0.001*
Glare test	binocular	-0.069	>0.05	-0.010	>0.05	0.022	>0.05

Frequencies of reported scotomas and distortion are given in Table 8. Mann Whitney tests were carried out to compare MacDQoL scores in those who did and did not report binocular distortion or scotomas within 10° of vision. A Bonferroni correction was applied (six tests, p <0.0083 accepted). None of the MacDQoL scores distinguished between those who did and did not report distortion, but compared with those who did not have binocular scotomas, those who did reported poorer present QoL (means [s.d.]: yes = 0.56 [1.21], no = 1.00 [1.09], U = 1728, p = 0.037), poorer MD-specific QoL (means [s.d.]: yes = -2.44 [0.79], no = -2.03 [0.88], U = 1607, p = 0.007) and lower AWI scores (means [s.d.]: yes = -4.73 [2.04], no = -3.10 [2.02], U = 1244, p < 0.001) The MD-specific QoL overview item and the AWI score comparisons remained significant after applying the Bonferroni correction.

**Table 8:** Frequencies (and valid %) of reported scotomas and distortion in each eye and with binocular vision

	Scotoma		Distortion	
	Yes (valid%)	No (valid%)	Yes (valid%)	No (valid%)

Right eye	80 (52.3)	73 (47.7)	68 (44.4)	85 (55.6)
Left eye	75 (48.7)	79 (51.3)	50 (32.7)	103 (67.3)
Binocular	39 (25.7)	113 (74.3)	58 (37.9)	95 (62.1)

### Open-ended question

In response to the final, open-ended question, 'Does MD affect your quality of life in any ways that have not been covered by the questionnaire?', 56 people answered 'yes'. Those people stated one or more ways in which MD affected their QoL. In most cases, the statements were covered by items in the MacDQoL. Sixteen mentioned reading, 11 hobbies, 6 getting out and about and 7 mentioned driving specifically. Seven people mentioned not being able to recognise people, which may be related to *friends and social life *or to *people's reaction*, but it may not be fully encompassed by either item. Five people said they were frustrated by MD. Frustration might be caused by many aspects of living with MD, including items in the MacDQoL, such as *time taken, mishaps and losing things, household tasks, personal affairs *among others. There was no clear case for needing additional items.

### Non-attenders

Five people completed the interview but subsequently did not attend the vision assessment. Mean MacDQoL scores (and s.d.s) for those people were: present QoL = 1.00 (1.22); MD-specific QoL overview = -2.4 (0.89); AWI = -3.13 (3.1). There were no significant differences in the MacDQoL scores between attenders and non-attenders (p's > 0.05).

## Discussion

A total of 156 people completed both the telephone interview and the vision assessment. This was 70% of those initially approached, representing a good response rate, particularly for this elderly population. The excellent completion rate of MacDQoL items (99.85%) far exceeds the 75% obtained with utility measures [33] and it indicates that the MacDQoL is a questionnaire that is acceptable to respondents.

The wide individual variation in the ratings of impact and of importance in the MacDQoL confirms that an individualised measure is needed. Weighting impact scores by importance ratings further refines the validity and investigative qualities of the measure. The fact that only three item means remained in the same rank order of impact once importance ratings had been incorporated shows that incorporating importance scores has a noticeable effect on QoL domain scores even at the level of group means and individual scores are markedly affected by weighting with importance scores.

The high reported negative impact on MD-specific domains such as *independence*, *personal affairs *and *do for others *suggests that the condition-specific measure will be more sensitive than a generic QoL measure, as it investigates aspects of life that are particularly impacted by MD and these are not included in many, if any, generic measures.

The 26-item MacDQoL was a long questionnaire and the removal of three redundant items will reduce the burden involved in its completion. Their meaning is encompassed in items retained in the questionnaire. A fourth item, *finances*, also appeared to be a candidate for removal, with a low impact rating, the lowest weighted impact score and a small reduction to the internal consistency of the scale. Also, it did not load well in the forced single-factor analysis. However, at the time of the study, the currently favoured treatment for focal wet MD, photodynamic therapy, was not available free of charge through the National Health Service and, for those who elected to have the treatment, the financial burden was considerable. The mean weighted impact score for *finances *masked considerable individual variation, suggesting it would be inappropriate to remove the item. If the MacDQoL is used in countries where payment for treatment is also the norm, the *finances *item will be salient. MD can also affect finances in other ways, with extra costs being incurred for work such as dressmaking, housework and house maintenance, which people with MD may have undertaken themselves when they had good eyesight. For some, there may be an improvement in finances due to an entitlement to disability allowances for severely visually impaired people. In the present study one person reported that his financial situation would be worse if he did not have MD.

Our preference was for a single factor, since a single score is easier to use in both research and clinical contexts. Principal components analysis was carried out to investigate the possibility of a strong multi-factor structure. Factors should be not only mathematically but also conceptually distinct and they should form logical rather than apparently arbitrary groups. There was some evidence of logical grouping in the analyses but it was not convincing for all items. In addition, when items were removed during the item reduction process, the factor structure did not withstand these changes, indicating that the factor structure was not stable. The forced single-factor analysis showed that all items except *work *and *finances *loaded well together and demonstrated a good single-factor structure, supporting the use of an overall average weighted impact score. The single factor structure improved further with the removal of *work *from the scale. Since the *work *item was applicable to so few people and there was little variability in the scores, this had an adverse effect on the cohesiveness of the scale. However, the item is likely to show high impact and importance for those who do work, and so it is important to retain the item in the questionnaire for scoring separately. The factor structure of the MacDQoL will be revisited at a later date using longitudinal data to ensure that sensitivity to change over time is not better measured using subscales.

The Cronbach's alpha of 0.944 for the 22-item scale indicates high internal consistency reliability. An alpha of at least 0.8 is regarded as adequate for group comparisons but for clinical work, with individual patients, an alpha of 0.9 is regarded as a minimum [34]. Together with the single-factor analysis, the reliability coefficient offered considerable support for combining the MacDQoL items in a single scale. Item-total correlations were also encouraging, ranging from 0.33 to 0.80. The alpha-if-item deleted figures showed that items are similar in their effect on alpha and these data did not offer clear evidence for the exclusion of any particular items. The high Cronbach's alpha, however, suggested that other items could be removed without detriment to the scale properties. It may be useful to consider further the weighted item scores and assess the impact of removing those with low weighted impact. Nevertheless, as seen with reference to the *finances *item, there may be good reasons for retaining some items even though their weighted impact scores are low.

Correlations between the MacDQoL AWI and the two overview items were moderate, with a higher correlation between the AWI and the MD-specific QoL item, as expected. The magnitude of the correlation, 0.58, which is markedly less than the 0.7 required to indicate minimum equivalence [35], indicates that the MD-specific QoL item is no substitute for the AWI score.

Investigations of the relationships between the MacDQoL scores and the scores on vision measures suggested that the questionnaire has construct validity since, before Bonferroni correction, 29 of the 36 associations investigated were significant (p < 0.05), and 20 of these remained significant after correction for familywise error. The associations that remained significant were, for the large part, those that were expected to show the strongest relationships. Overall, measures of better eye and binocular vision were more strongly associated with the MacDQoL variables than measures of worse eye vision. This is to be expected, since visual ability is largely a function of the better eye and binocular function will be mainly dependent on function in the better eye. The MacDQoL demonstrates that MD has considerable negative impact on many aspects of QoL, particularly independence, leisure activities, ability to deal with personal affairs and mobility. The more severe the visual impairment due to MD, the greater is the negative impact of the condition on QoL.

The AWI score showed stronger correlations with vision measures than the MD-specific QoL overview item. The AWI is a combination of scores from domains that participants are specifically asked to consider, and the variable thus offers a more systematic assessment of the impact of MD on QoL than does the overview item. We would expect it to show a stronger association with the vision measures than the overview item. Nevertheless, the MD-specific QoL overview item may be sufficiently sensitive to be considered for use alone, for example, for audit purposes.

The present QoL item was less strongly associated with vision measures than were the AWI score and, to a lesser extent, the MD-specific QoL overview item and this finding was anticipated since, in assessing present QoL, individuals consider many factors other than the impact of MD on QoL. The present QoL item did show significant associations with a number of measures (particularly measures of binocular vision), even after the Bonferroni correction, and this demonstrates the extent of the damage done to QoL by vision loss resulting from MD.

The only vision test with which the MacDQoL scores did not show any relationship was the Eger glare test. The usefulness of the Eger stressometer in assessing the effect of glare on people with MD has yet to be established but the present data suggest little if any impact on QoL of glare as measured by this new method of assessment.

Bradley et al [17] noted that items with an N/A option presented challenges when carrying out psychometric evaluation of QoL measures. The procedure employed here for dealing with missing data caused by N/A options was used by Bradley et al [17] in order to retain sufficient data to carry out the psychometric analyses and to make best use of the available data. Both in the earlier study [17] and in the present study, using zero to replace N/A, with listwise deletion and treating N/A as missing with pairwise deletion yielded similar results. In subsequent data analysis, however, items that were N/A were excluded from the weighted mean scores. If there were no N/A options available, participants would be likely to use 'the same' responses to the impact scale and score zero for any item that was not relevant to them and this would artificially lower the AWI score. The N/A option is a feature of this and other measures based on the ADDQoL that, in addition to weighting items by importance, makes the instruments individualised measures.

The open-ended question, which asked if there were any ways in which MD affected QoL that were not covered by the questionnaire, solicited 56 responses. However, in the majority of cases, people commented on aspects of life that had in fact been covered by the questionnaire. People often made comments at this point to emphasize the things that were most important to them, such as reading and driving. Five people mentioned frustration. Frustration has not been stated explicitly in any MacDQoL items, but it is implicit in most of them. Seven people mentioned not recognising people and, whereas there are a number of items that refer to interacting with others, this problem may not adequately be addressed by any of them. It will be monitored in future work.

There was no difference in the MacDQoL scores of those who did and did not attend the vision assessment. The large discrepancy in the number of people in each group would make a significant difference unlikely, but most of those who did not attend were either ill on the day or unable to travel due to bad weather. It is not surprising, therefore, that the MacDQoL scores are similar.

The MacDQoL was originally designed for self-completion. Due to the anticipated severity of visual impairment of some people in this sample the questionnaire was administered by telephone. A mixture of completion methods was decided against since previous research has shown that people report less negative impact of MD on QoL during telephone interviews than when self-completing the MacDQoL [22]. Using a single administration method ensured that real differences in QoL due to severity of MD were not masked by biases caused by methodology.

The sample participating in this study may differ from the MD population as a whole in several ways. All but six people had MD in both eyes. This probably reflects the fact that participants were selected from an ophthalmologist's clinic records. In the general population, many cases of dry MD in one eye alone remain undiagnosed, or do not get referred to a specialist, as there is currently no treatment available. The full range of severity of MD, wet and dry, was represented in the sample ensuring that the suitability of the MacDQoL was assessed for representatives of the MD population as a whole. The AWI score may be relatively high due to the sample generally having more severe MD than in the MD population as a whole, but this would not affect the psychometric properties of the questionnaire in any way or its usefulness for people with milder MD.

Participants completed the MacDQoL by telephone interview and the methodology precluded seven people who were originally selected to be invited to participate but who did not have a telephone. A number of people who were also originally selected to be invited to participate were not contactable because contact details, including telephone numbers, were not up to date. It was not possible to ascertain whether those people had moved, no longer had a telephone or were deceased. Hearing impairment would also have precluded people from participating, but no one who was approached gave hearing impairment as a reason for not wishing to take part.

## Conclusion

The MacDQoL individualised measure of the impact of MD on quality of life has been shown to have good psychometric properties. By inviting participants to rate both the impact of MD on domains of life and the importance of those domains to QoL and by providing 'not applicable' options it allows for a more individualised investigation of the impact of MD on QoL than is possible with visual function questionnaires. Excellent completion rates attest to the acceptability of the measure. The MacDQoL has been shown to have good face and construct validity with expected associations with visual function, particularly when assessed binocularly or with the better eye. The measure demonstrates that MD has a considerable negative impact on many aspects of life and on quality of life per se. The MacDQoL is now ready for use in clinical trials, routine clinical care and the evaluation of service provision.

## Authors' contributions

JM participated in the design of the study and in coordination of the research, carried out telephone interviews, performed statistical analysis and drafted the manuscript. JW participated in the design of the study, carried out vision assessments and prepared the vision assessment data for analysis. AW participated in the design of the study, led the writing of the protocol and application for ethical approval and participated in coordination of the research. SJA participated in the design of the study, carried out vision assessments and, with JW, prepared the clinical data for analysis. CM carried out telephone interviews and contributed to the selection of redundant items. Tf participated in the design of the study and advised on clinical matters in the selection of participants. MR participated in the design of the study and carried out vision assessments. WA participated in the design of the study, selected participants from his clinics and oversaw the recruitment of participants and the vision assessment clinics. CB, the lead investigator, conceived of the study, participated in its design and oversaw progress of the work. All authors read and approved the final manuscript.

## Copyright of MacDQoL questionnaire

For access to and permission to use the MacDQoL questionnaire, contact the copyright holder, Clare Bradley PhD, Professor of Health Psychology, Health Psychology Research, Royal Holloway, University of London, Egham, Surrey, TW20 0EX: c.bradley@rhul.ac.uk.
